# Design of Network-on-Chip-Based Restricted Coulomb Energy Neural Network Accelerator on FPGA Device

**DOI:** 10.3390/s24061891

**Published:** 2024-03-15

**Authors:** Soongyu Kang, Seongjoo Lee, Yunho Jung

**Affiliations:** 1 School of Electronics and Information Engineering, Korea Aerospace University, Goyang 10540, Republic of Korea; tnsrb18@kau.kr; 2 Department of Electrical Engineering, Sejong University, Seoul 05006, Republic of Korea; seongjoo@sejong.ac.kr; 3 Department of Convergence Engineering of Intelligent Drone, Sejong University, Seoul 05006, Republic of Korea; 4 Department of Smart Air Mobility, Korea Aerospace University, Goyang 10540, Republic of Korea

**Keywords:** field-programmable gate array, internet of things, network-on-chip, restricted coulomb energy neural network

## Abstract

Sensor applications in internet of things (IoT) systems, coupled with artificial intelligence (AI) technology, are becoming an increasingly significant part of modern life. For low-latency AI computation in IoT systems, there is a growing preference for edge-based computing over cloud-based alternatives. The restricted coulomb energy neural network (RCE-NN) is a machine learning algorithm well-suited for implementation on edge devices due to its simple learning and recognition scheme. In addition, because the RCE-NN generates neurons as needed, it is easy to adjust the network structure and learn additional data. Therefore, the RCE-NN can provide edge-based real-time processing for various sensor applications. However, previous RCE-NN accelerators have limited scalability when the number of neurons increases. In this paper, we propose a network-on-chip (NoC)-based RCE-NN accelerator and present the results of implementation on a field-programmable gate array (FPGA). NoC is an effective solution for managing massive interconnections. The proposed RCE-NN accelerator utilizes a hierarchical–star (H–star) topology, which efficiently handles a large number of neurons, along with routers specifically designed for the RCE-NN. These approaches result in only a slight decrease in the maximum operating frequency as the number of neurons increases. Consequently, the maximum operating frequency of the proposed RCE-NN accelerator with 512 neurons increased by 126.1% compared to a previous RCE-NN accelerator. This enhancement was verified with two datasets for gas and sign language recognition, achieving accelerations of up to 54.8% in learning time and up to 45.7% in recognition time. The NoC scheme of the proposed RCE-NN accelerator is an appropriate solution to ensure the scalability of the neural network while providing high-performance on-chip learning and recognition.

## 1. Introduction

The internet of things (IoT) technology interconnects various computing devices to be aware of and interact with their external environment. This enhances users’ quality of life and improves efficiency and sustainability in day-to-day activities [1]. In IoT systems, sensors collect crucial information from the external environment, enabling meaningful decision making [2]. Sensor applications in IoT systems are actively being researched in a variety of fields such as real-time processing for industrial applications, healthcare, and scientific activities [3]. Edge-based computing is preferred for these applications to meet modern requirements for latency, mobility, and energy efficiency [4].

Artificial intelligence (AI) technologies offer analytics for IoT sensor data across various sensor applications [5,6,7,8]. Deep neural networks (DNNs), such as the convolutional neural network (CNN), recurrent neural network (RNN), and long short-term memory (LSTM) have achieved high classification accuracy in many applications. However, as DNNs become more complex, their computational cost increases extremely [9]. The enormous model size also poses challenges for implementation on resource-constrained edge devices because of the high hardware resource consumption, such as memory usage and floating point operations. In addition, the network structure of the DNNs, such as the number of neurons and layers, is optimized depending on the specific application. Therefore, when the application changes or new learning data are introduced, adjustments or the relearning of the network becomes necessary. The complexity of DNN algorithms makes them challenging to learn on edge devices, requiring skilled technicians for network adjustment. Consequently, DNNs are not well-suitable for achieving low-latency processing on resource-constrained edge devices.

In contrast, a restricted coulomb energy neural network (RCE-NN) generates neurons as needed during the learning process. This allows the network structure to adjust flexibly, making it well-suited for various sensor applications on edge devices [10]. In addition, the computation for learning and recognition in RCE-NNs involves simultaneously calculating the distance between the input feature vector and learned parameters and comparing the outputs of activated neurons. Given this low-complexity computational structure and parallelism, the RCE-NN was implemented as a hardware accelerator capable of real-time processing [10,11,12]. The authors of [10] proposed an improved RCE-NN learning algorithm that can achieve a high classification accuracy with fewer neurons than the basic RCE-NN algorithm. They presented the results of a high-speed and low-power very-large-scale integrated circuit (VLSI) implementation. In [11], the dynamic time warping (DTW) algorithm, which is effective for analyzing time series data, was applied to the distance calculation algorithm. Accordingly, they implemented a hand gesture recognition (HGR) system based on inertial measurement unit (IMU) sensors with a high classification performance and presented the hardware implementation results. The authors of [12] proposed an RCE-NN-based face recognition system using CMOS image sensors and implemented it as a hardware accelerator. Operating at a 62.5 MHz clock frequency, the system takes 18 ms to recognize an image and achieved high accuracy on four datasets. Nevertheless, in previous RCE-NN accelerators, the input feature vector and control signals are broadcast to all neurons within the parallel network structure, leading to elevated fan-in/out requirements for circuitries connected to neurons. Thus, previous RCE-NN accelerators have limited scalability with the number of neurons to prevent system performance degradation in terms of the operating frequency.

The network-on-chip (NoC) scheme for the complex communication requirements provides scalable and flexible communication for massively parallel systems [13]. NoCs have overcome scalability limitations by providing a flexible communication structure with high performance [14,15]. In addition, the authors of [16,17] present that unlike the shared bus topology, which cannot provide a dense interconnection for neural networks, the NoC scheme can provide high scalability with low power consumption by eliminating the one-to-one correspondence between nodes, in addition to reconfigurability. As such, the NoC scheme ensures network scalability and efficient communication, making it suitable for implementing high-performance accelerators for AI-based IoT technologies. Therefore, we propose a novel NoC-based RCE-NN accelerator to effectively address scalability issues from previous implementations. The topology and routers are designed to be specific for the RCE-NN. As a result, the fan-in/out was fixed at a reasonable level, overcoming the problem of a sharp drop in the maximum operating frequency with the number of neurons. Accordingly, the proposed RCE-NN accelerator can perform real-time processing on edge devices for various sensor applications. The proposed RCE-NN accelerator was implemented on a field-programmable gate array (FPGA) and verified with two datasets, demonstrating accelerations of up to 54.8% in learning time and up to 45.7% in recognition time.

The remainder of this paper is organized as follows: In Section 2, we briefly introduce the learning and recognition algorithms of the RCE-NN and analyze previous RCE-NN accelerators. In Section 3, we discuss the NoC topology suitable for the RCE-NN. In Section 4, we describe the proposed RCE-NN accelerator. In Section 5, we present the results of implementating the proposed RCE-NN accelerator on an FPGA and the acceleration effect. Finally, in Section 6, we conclude this paper.

## 2. Restricted Coulomb Energy Neural Network

**Figure 1:**
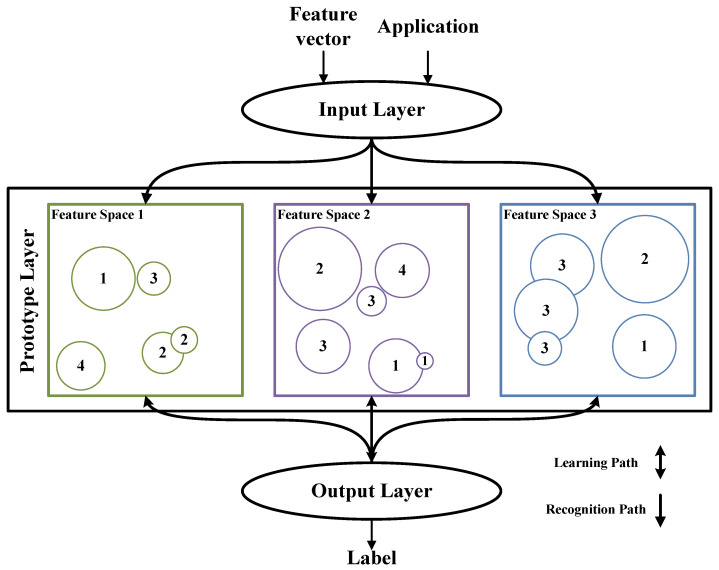
Example of an RCE-NN structure for three applications.

The RCE-NN is a supervised machine learning algorithm designed to learn and recognize the feature regions of input vectors [18]. Each neuron generated during the learning process constitutes a feature region associated with a label. Because the feature space of a neuron is organized by application, RCE-NNs can be used to recognize or learn multiple applications independently. Figure 1 illustrates an example of an RCE-NN structure comprising three feature spaces from three different applications. In Figure 1, each application’s feature vector is two dimensional, the neuron’s feature region is represented as a circle, and the numbers within the circles represent labels. The number of feature spaces is equal to the number of applications, and the dimensions of the feature vector and the shape of the feature region are chosen for each application. The RCE-NN consists of an input layer, a prototype layer, and an output layer. The input and output layers are connected to all the neurons generated in the prototype layer. The input layer transfers the input feature vector to the neurons in the prototype layer. Neurons in feature spaces different from the input application remain inactive. However, neurons in the feature space of the input application, whose input feature vector falls within the feature region, are activated. The activated neurons calculate the distance between the center point of the feature region and the input feature vector, transferring the result to the output layer. The output layer then generates and adjusts neurons or outputs the recognition results.

As shown in Figure 1, feature regions can only overlap with feature regions that have the same label to avoid confusion during the recognition process. This means that every coordinate in the feature space has either one label correspondence or no label correspondence. The arrows connecting the output layer and prototype layer are represented in both directions for feedback from the output layer during the learning process.

$x^{A}$, defined as in Equation (1), is the input feature vector for learning with application A and label l:

(1) $$x^{A}={\left[f^{1},f^{2},\ldots f^{K},l\right]}^{A}={\left[f,l\right]}^{A}$$

The vector f represents a feature vector. K is the dimensions of the feature vector. If no neuron is active during the learning process, a neuron is generated by using the $x^{A}$ information. The *i*-th neuron generated by $x^{A}$ during the learning process is defined as in Equation (2):

(2) $$p_{i}^{A}={\left[c_{i}^{1},c_{i}^{2},\ldots c_{i}^{K},r_{i},l\right]}^{A}={\left[c_{i},r_{i},l\right]}^{A}$$

The vector $c_{i}$ and $r_{i}$ represent the center point and radius of the feature region, respectively. The vector $c_{i}$ is equal to f in $x^{A}$. If any of the neurons activated during the learning process has a different label than the input vector, the radius of the neuron is reduced to the distance between f and $c_{i}$. The distance calculation method is performed by using an appropriate method, such as the L1 norm, L2 norm, or sup norm, depending on the application. During the recognition process, the output layer generates the label l of the neuron closest to f among the activated neurons as the recognition result. This way, the RCE-NN is suitable for various sensor applications by segmenting the feature space according to the application. Furthermore, simple computation enables edge-based computing for both learning and recognition.

### Previous RCE-NN Accelerators

**Figure 2:**
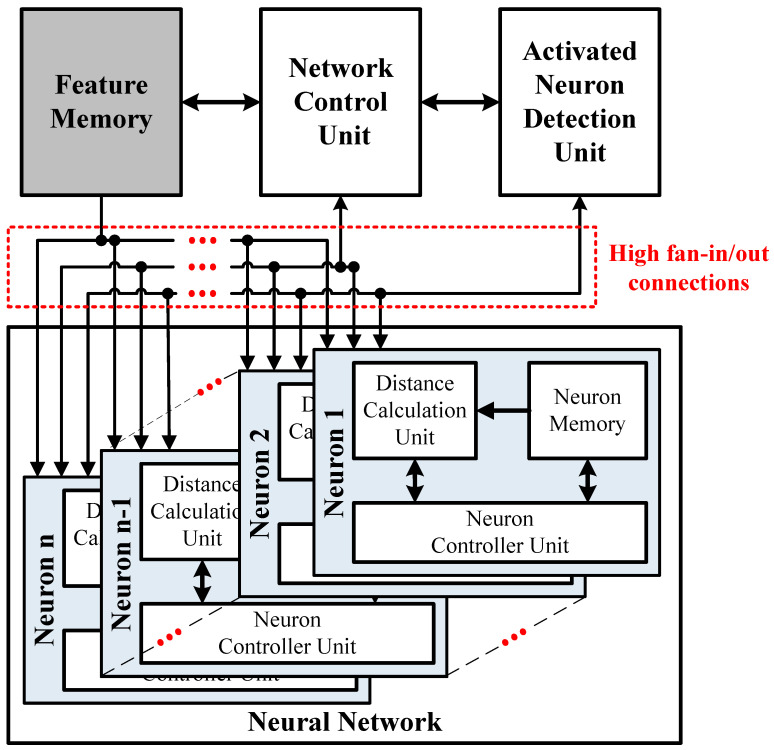
Overview of the previous RCE-NN accelerators.

The authors of [10,11] implemented the RCE-NN as hardware accelerators for sensor applications with real-time learning and recognition. Figure 2 shows an overview of the accelerator structure described in [10,11]. The neurons are connected in parallel with a feature memory, a network control unit (NCU), and an activated neuron detection unit (ANDU). The neurons receive feature vectors from the feature memory via broadcasting and transfer the computed information to the NCU and ANDU. In these previous RCE-NN accelerators, the neurons and circuitries connected to the neurons exchange a large number of signals in parallel. This results in a reduction in the maximum operating frequency due to high fan-in/out connections. Thus, an increase in the number of neurons will negatively affect the processing speed of the accelerator. Previous RCE-NN accelerators have limitations in their ability to perform real-time processing when a large number of neurons are required in applications.

## 3. NoC Topology for RCE-NN Accelerator

To overcome the limitation of the broadcasting used by previous RCE-NN accelerators, we propose an NoC-based RCE-NN accelerator. The NoC architecture consists of cores and routers, providing scalability and flexibility to the network. In the NoC architecture, routers enable intercore communication between the source core and target core based on the NoC topology. The proposed RCE-NN accelerator’s communication structure comprises neuron cells, cores, and routers. The neuron cells in the proposed RCE-NN accelerator, similar to the neurons in the previous RCE-NN accelerators, serve as processing elements (PEs) responsible for computing distances and transferring the results to the core. The core is responsible for processing the information received from the neuron cells and learning them based on the RCE-NN algorithm. Routers enable communication between cores, as well as between cores and neuron cells.

Choosing an optimal NoC topology is a crucial step in NoC architecture design [19]. The RCE-NN requires multicasting communication to transfer input feature vectors to multiple neuron cells. To ensure network scalability, it is essential to maintain an appropriate fan-in/out, irrespective of the number of neuron cells. The hierarchical topology is suitable for interconnecting homogeneous cores [20] with a low traffic hop distance—indicating the number of nodes needed to forward data to a target core [21]. The star topology offers a high-speed communication performance at low network traffic, characterized by short traffic hop distances [22]. Furthermore, it simplifies the process of adding and removing cores. The hierarchical–star (H–star) topology is a topology that combines the hierarchical and star topologies. The H–star topology is energy and cost efficient and provides an efficient way to utilize the multicast communication of many neurons [19]. In addition, because the number of nodes connected to the routers in each hierarchy is fixed, the fan-in/out requirements are fixed, keeping the maximum operating frequency at a constant level. In other words, the scalability of the H–star topology-based NoC architecture includes flexible circuit configuration and a fixed path delay. These advantages have led to the use of H–star topologies in chip multiprocessor (CMP) systems and image processing as well [23]. Hence, in this paper, we employ the H–star topology as the NoC topology for the RCE-NN, given its suitability. The H–star topology enables the proposed RCE-NN accelerator to add neurons freely and overcomes the limitation of scalability.

## 4. Proposed RCE-NN Accelerator Based on NoC

**Figure 3:**
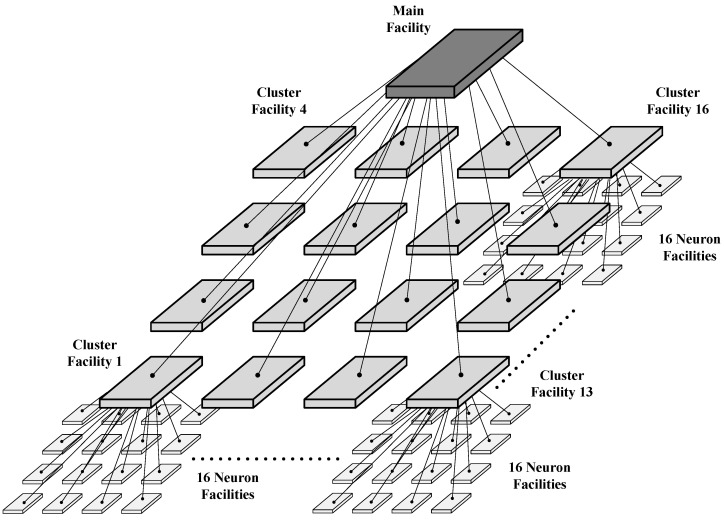
Architecture of the proposed RCE-NN accelerator.

**Figure 4:**
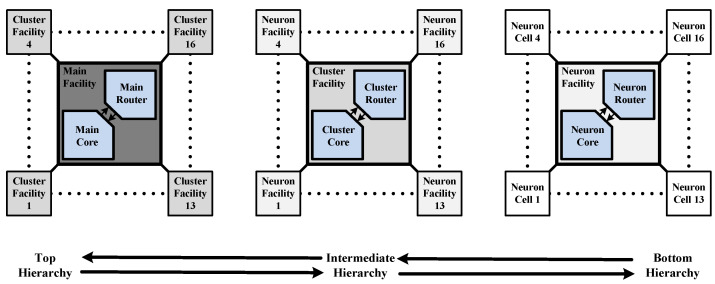
Hierarchy of the proposed RCE-NN accelerator.

Figure 3 shows the NoC architecture based on the H–star topology of the proposed RCE-NN accelerator. Figure 4 shows the hierarchy of the proposed RCE-NN accelerator. In the proposed RCE-NN accelerator, three levels of facilities manage sub-facilities or neuron cells through a hierarchical topology. Each facility employs a star topology to group 16 child nodes. The router at each facility handles on-chip communication traffic based on RCE-NN algorithms. The main facility manages the cluster facilities, the cluster facilities manage the neuron facilities, and the neuron facilities manage the neuron cells.

The proposed RCE-NN accelerator operates with data flows consisting of top-down and bottom-up directions. The top-down data flow involves transmission data packets from the main core of the top hierarchy to the neuron cells. When the main facility receives the input feature vector, it tracks the neuron cells that have the same application information as the input. The routing table of each facility determines the path to the neuron cells. In contrast, the bottom-up data flow operates in the opposite direction. Facilities in the intermediate and bottom hierarchies receive information from the 16 child nodes, generate a single data packet, and then forward it to the parent nodes. This process is repeated according to the RCE-NN algorithm, which performs learning and recognition tasks.

### 4.1. NoC Design for the Proposed RCE-NN Accelerator

**Figure 5:**
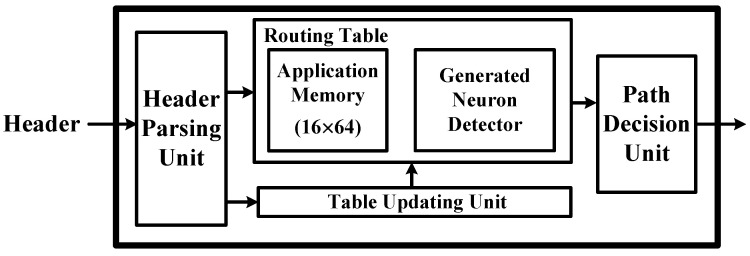
Routing decision unit of main facility and cluster facility.

The NoC designs for the proposed RCE-NN accelerator are summarized as follows: Data flow control is managed through headers and RCE-NN-specific routing tables that store the application information of child nodes. The routing algorithm utilizes a distributed routing approach, where each node activates paths to child nodes that have the same application information as the header, utilizing its routing table. The routing decision unit (RDU) of the main and cluster facilities are shown in Figure 5. The header parsing unit transfers the application information from the header to the routing table. This application information serves as the address for the application memory. The width of the application memory is 16 bits, corresponding to the number of child nodes managed by the facility. Each bit represents the presence or absence of child nodes having the same application information as the header. Consequently, the routing to child nodes is determined by reading the application memory based on the application information in the header. Since each facility only manages application information for its child nodes, this approach effectively solves the problem of managing a large number of nodes with distributed routing algorithms [24]. The remaining components of the RDU consist of a generated neuron detector for information about the network structure and a table-updating unit based on the learning results. The RCE-NN-specific routing algorithm efficiently transfers data packets exclusively to neuron cells that possess matching application information.

The switching techniques employed in the proposed RCE-NN accelerator include the circuit-switched (CS) method and the wormhole method [25,26]. The learning and recognition process initiates with a top-down data flow, where each router utilizes headers and routing tables to determine sub-paths for sending and receiving data packets. The path decision unit maintains the determined paths. In other words, during the initial top-down data flow, each router determines the path to the child node by using the wormhole method, which is then maintained by using the CS method until the completion of the algorithmic behavior. Nodes and neuron cells are interconnected in a one-to-many configuration, although a one-to-one configuration can also be used depending on the number of neurons to be implemented. The links connecting the cores are exclusively dedicated to the two cores without being shared by the other cores. Additionally, there is a delay due to one level of registers per layer in the communication process. The network interface (NI) associated with the router is specifically designed for handling data packets for the RCE-NN. Consequently, the proposed RCE-NN accelerator employs NoC design techniques optimized for the communication structure of the RCE-NN, enabling efficient intercore communication.

**Figure 6:**
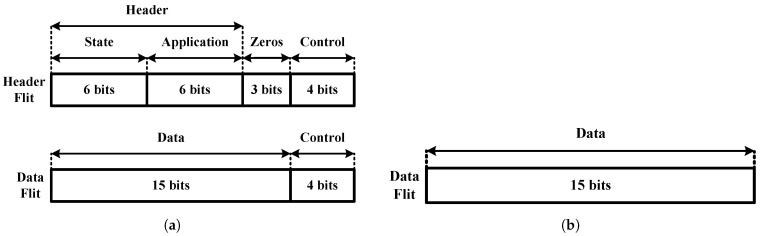
Data packets: (**a**) top-down data packet; (**b**) bottom-up data packet.

Figure 6 shows the format and field composition of the data packets used in the on-chip communication of the proposed RCE-NN accelerator. The top-down data packet is divided into two parts: the header flit and the data flit, as shown in Figure 6a. The header flit consists of the header and control fields. The header field contains the state information from the main core’s finite state machine (FSM) and the application information. On the other hand, the data flit consists of the data and control fields. The core transfers the data fields to the neurons, which include the feature vector or maximum radius information. In contrast, the bottom-up data packets solely employ a data flit composed of data fields, as shown in Figure 6b.

### 4.2. Facilities

**Figure 7:**
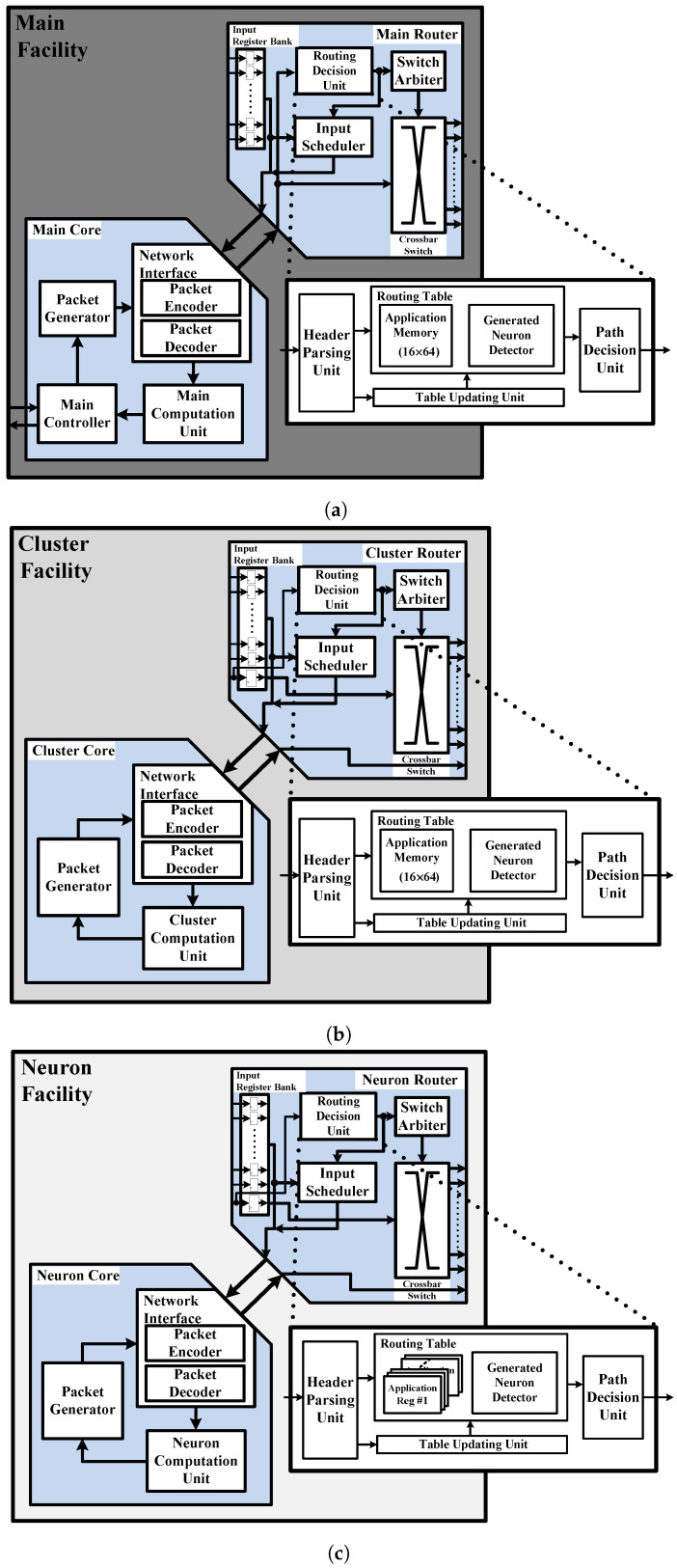
Block diagrams of the facilities: (**a**) main facility; (**b**) cluster facility; (**c**) neuron facility.

Figure 7 shows block diagrams of the hierarchical facilities of the proposed RCE-NN accelerator. Figure 7a shows a block diagram of the main facility of the proposed RCE-NN accelerator. The proposed RCE-NN accelerator includes a main facility of the top hierarchy. The main facility is responsible for managing the overall behavior of the RCE-NN algorithm and allocating traffic to the network by using the main core and main router. The main core consists of the main computation unit, main controller (MCR), packet generator (PG), and NI. The main computation unit identifies the neuron indicated by the recognition results and decides whether to adjust the network. The MCR decides the next action based on the FSM and transfers the necessary information for generating the packet data to the PG. The main router provides on-chip communication by using an RCE-NN-specific routing algorithm and switching techniques. It consists of the input register bank, RDU, switch arbiter, input scheduler, and crossbar switch. The switch arbiter and crossbar switch manage the input and output of the data packets. The input register bank receives and stores data transferred from the child nodes, while the input scheduler manages the bottom-up data flow.

The data flow within each facility is as follows: The MCR receives the input feature vector from the external circuitry and generates information to be sent to the neuron cells. The PG constructs the packet data by using the generated information. Data passing through the NI are then transmitted to the cluster facilities through the RDU and crossbar switch. Similarly, the cluster and neuron facilities forward the information to the neuron cells. The computed results from the neuron cells are subsequently sent back to the main facility through the intermediate and bottom hierarchies facilities. Once all the computed data from the active neuron cells have arrived at the main facility, the input scheduler finalizes the bottom-up data flow and transfers the data stored in the input register bank to the main core. The main computation unit performs computations for learning and recognition and transfers the information to the MCR. Finally, upon completing the operation, the MCR outputs the learning and recognition results.

The block diagrams for cluster and neuron facilities are shown in Figure 7b,c. The facilities of each hierarchy provide efficient data communication between their parent and child nodes. Except for the RDU of the neuron router and the input register bank, the units in each hierarchy perform the same roles as the units in the main facility described earlier. The routing table of the neuron router only stores application information for the 16 neuron cells. Hence, the routing table consists of 16 registers, each with six data bits. Unlike the main router, the input register bank of the cluster router and neuron router receive data packets from the parent node. As a result, they consist of 16 registers to store the data received from the child nodes, along with one register to store data received from the parent node. Each hierarchy facility in the proposed RCE-NN accelerator manages up to 16 child nodes. With the hierarchy divided into three levels, a maximum of 16 × 16 × 16 = 4096 neuron cells can be implemented.

### 4.3. Neuron Cell

**Figure 8:**
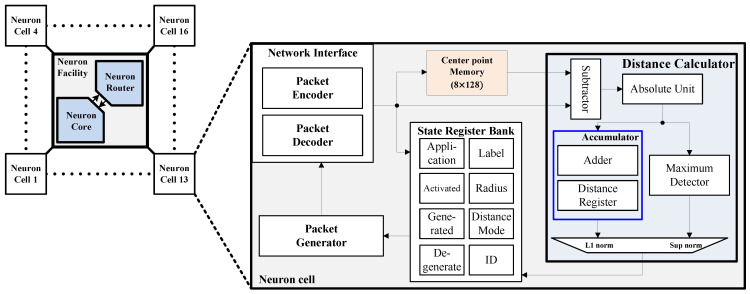
Block diagram of the neuron cell.

Figure 8 shows a block diagram of the neuron cell connected to the neuron facility. The NI transfers information in the data packet to various components, such as the center point memory, distance calculator, and state register bank. This transmission is based on the neuron cell’s state and control fields. The distance calculator performs calculations to determine the distance between the center point and input features by using a distance calculation algorithm. Once the neuron cell’s state update and distance calculation are complete, the PG generates packet data and sends them to the neuron router via the NI. The degenerate register indicates that the neuron cell has a radius smaller than the minimum required radius and should be reduced during the learning process.

## 5. Hardware Implementation

### 5.1. Implementation Results

The proposed RCE-NN accelerator is designed by using the Verilog 2001 hardware description language (HDL) and implemented on Xilinx’s Zynq Ultrascale+ ZCU104, which includes 504 K system logic cells, 1728 DSP slices, and 38 Mb memory [27]. For the implementation and verification, the software is responsible for generating input feature vectors containing information such as learning or recognition, application, and labels from the sensor dataset. It transfers them to the proposed RCE-NN accelerator and initiates its operation. The hardware device implements the proposed RCE-NN accelerator and verification system on the FPGA and performs operations of the RCE-NN on the input feature vectors. Table 1 shows the hardware usage and maximum operating frequency in relation to the number of neurons for both the previous RCE-NN accelerator and the proposed RCE-NN accelerator. For comparison, the previous RCE-NN is also implemented on an FPGA. With 512 neurons, the proposed RCE-NN accelerator shows a 0.6% increase in look-up tables (LUTs) usage and a 13.0% increase in flip-flops (FFs) usage compared to the previous accelerator. These increases are attributed to the routing algorithm and hierarchical communication architecture. Additionally, there is approximately a 0.6% increase in memory usage due to the inclusion of application memory in the routing table. Although the NoC architecture slightly increases hardware usage, it significantly boosts the maximum operating frequency by approximately 126.1%. In the proposed RCE-NN accelerator, the maximum operating frequency decreases slightly as the number of neurons increases. This decrease is attributed to the increased utilization of hardware resources, which in turn complicates the configuration of the implemented FPGA logic.

According to the H–star NoC architecture and RCE-NN operation, communication between nodes occurs solely between the main facility and neuron cells, which is relayed by the cluster and neuron facilities. Therefore, the node-to-node delay varies depending on the number of features. For example, for a gas dataset with 128 features, the overall node-to-node delay for data flow in both directions is 128 + 15 cycles for the learning process and 128 + 49 cycles for the recognition process.

### 5.2. Verification and Evaluation

**Figure 9:**
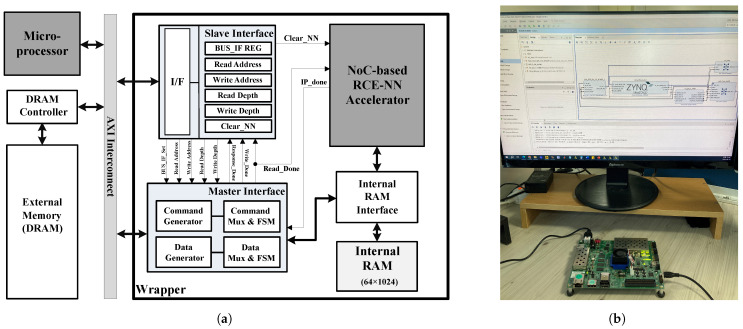
Verification environment: (**a**) system configuration; (**b**) verification with FPGA device.

Figure 9 shows the FPGA verification environment of the proposed RCE-NN accelerator. For FPGA verification, the configured system shown in Figure 9a was used. In this system, the proposed RCE-NN accelerator communicates with external circuitries through an advanced extensible interface (AXI) interconnection. The proposed RCE-NN accelerator interacts with the microprocessor via the slave interface and with the DRAM through the master interface. The master interface handles the transfer of the input feature vectors from the DRAM to the internal RAM and the transfer of learning or recognition results from the internal RAM to the DRAM. Figure 9b shows the verification environment on an FPGA device.

In this environment, we employed two datasets for gas sensors and Libras Movement (LM) to verify the proposed RCE-NN accelerator and evaluate its acceleration effects [28,29]. The gas dataset contains 13,910 instances collected by 16 chemical gas sensors for discriminative analysis across six gases. The LM dataset contains 360 instances representing 15 different gestures in Libras, the Brazilian sign language. These datasets are represented by 128 and 90 features, respectively.

To evaluate the acceleration effect, we measured the processing time with the maximum operating frequency for the number of neurons. Table 2 presents the processing time and acceleration effects for the gas dataset, while Table 3 presents the corresponding values for the LM dataset. With an increase in the number of neurons, the maximum operating frequency of the previous RCE-NN accelerator notably decreases, leading to a significant rise in the processing time. However, the proposed RCE-NN accelerator, while experiencing a slight reduction in the maximum operating frequency, yields a negligible increase in processing time. The proposed RCE-NN accelerator attains a notable 54.8% reduction in processing time compared to the previous RCE-NN accelerator, even with a marginal increase in the required cycles due to the NoC structure. To achieve high accuracy in classifying datasets with numerous instances, hundreds of neurons are necessary [10]. Thus, the proposed RCE-NN accelerator, demonstrating over 36% acceleration with hundreds of neurons, is well-suited for real-time processing on edge devices across a variety of sensor applications.

## 6. Discussion and Conclusions

In this paper, we propose an NoC-based RCE-NN accelerator that addresses the scalability limitation of the number of neurons for various sensor applications. The key aspect of the NoC architecture, the NoC topology, is chosen to be the H–star topology considering the characteristics of the RCE-NN. The proposed RCE-NN accelerator with an H–star topology efficiently manages a large number of neuron cells through hierarchical facilities. Each facility has a fixed number of connected nodes, ensuring fixed fan-in/out requirements regardless of the number of neurons. We implemented the proposed RCE-NN accelerator on an FPGA. In terms of FPGA implementation, the proposed RCE-NN accelerator with 512 neurons resulted in a hardware usage increase of 0.6% for LUTs and 13.0% for FFs compared to the previous RCE-NN accelerator. However, it demonstrated a 126.1% increase in the maximum operating frequency. As a result, despite a slight increase in hardware resources and processing cycles due to the NoC architecture, the processing time is reduced by up to 54.8%. The experimental results demonstrate that the proposed RCE-NN accelerator exhibits greater acceleration effects with a larger number of neurons compared to the previous RCE-NN accelerator. The NoC architecture of the proposed RCE-NN accelerator offers scalability across a wide range of sensor applications. By addressing the limitations of the previous RCE-NN accelerator, this paper extends the possibilities for developing RCE-NN accelerators for on-chip learning and recognition. In future work, we plan to design a system that integrates the proposed RCE-NN accelerator, sensor, and feature extractor and implement it on VLSI.

## Figures and Tables

**Table 1:** Comparison of the RCE-NN accelerator implementation results based on the number of neurons.

# Neurons	[10]				Proposed			
	# LUTs	# FFs	RAM (Bytes)	Max Frequency (MHz)	# LUTs	# FFs	RAM (Bytes)	Max Frequency (MHz)
16	3456	1910	2048	247.8	4166	2307	2304	338.5
32	7028	3820	4096	236.4	7739	4348	4352	338.9
64	14,238	7565	8192	234.3	14,707	8424	8448	322.2
128	27,923	14,664	16,384	212.8	28,661	16,570	16,640	306.4
256	56,438	29,211	32,768	146.2	56,559	32,877	33,024	301.3
512	112,030	58,076	65,536	132.6	112,714	65,623	65,920	299.8

**Table 2:** Processing time and acceleration effects for gas dataset at maximum operating frequencies.

# Neurons	Learning Process			Recognition Process		
	[10] (μs)	Proposed (μs)	Acceleration	[10] (μs)	Proposed (μs)	Acceleration
16	0.57	0.42	25.3%	0.58	0.52	10.0%
32	0.59	0.42	28.7%	0.61	0.52	14.3%
64	0.60	0.44	25.8%	0.62	0.55	10.7%
128	0.66	0.47	29.0%	0.68	0.58	14.6%
256	0.96	0.48	50.4%	0.99	0.59	40.4%
512	1.06	0.48	54.8%	1.09	0.59	45.7%

**Table 3:** Processing time and acceleration effects for Libras Movement dataset at maximum operating frequencies.

# Neurons	Learning Process			Recognition Process		
	[10] (μs)	Proposed (μs)	Acceleration	[10] (μs)	Proposed (μs)	Acceleration
16	0.41	0.31	24.8%	0.43	0.41	4.0%
32	0.43	0.31	28.1%	0.45	0.41	8.5%
64	0.44	0.33	25.1%	0.45	0.43	4.6%
128	0.48	0.34	28.4%	0.50	0.45	8.8%
256	0.70	0.35	50.1%	0.73	0.46	36.4%
512	0.77	0.35	54.5%	0.80	0.46	41.9%

## Data Availability

Data are contained within the article.
